# Guest editorial: Papers from the 18th joint workshop on Augmented Environments for Computer Assisted Interventions (AE‐CAI) at MICCAI 2024: Guest editors’ foreword

**DOI:** 10.1049/htl2.70000

**Published:** 2025-01-20

**Authors:** Cristian A. Linte, Ziv Yaniv, Elvis Chen, Simon Drouin, Marta Kersten‐Oertel, Jonathan McLeod, Duygu Sarikaya, Jiangliu Wang

**Affiliations:** ^1^ Biomedical Engineering and Imaging Science Rochester Institute of Technology Rochester USA; ^2^ National Institutes of Allergy and InfectiousDisease National Institutes of Health Bethesda USA; ^3^ Department of Electrical and Computer Engineering Western University London Canada; ^4^ Department of Software and IT Engineering École de Technologie Supérieure Montreal Canada; ^5^ Gina Cody School of Engineering and Computer Science Concordia University Montreal Canada; ^6^ Nvidia Inc. Santa Clara USA; ^7^ School of Computer Science University of Leeds Leeds UK; ^8^ Department of Mechanical and Automation Engineering Chinese University of Hong Kong Hong Kong China

## 1 WELCOME AND THANK YOU FOR ANOTHER SUCCESSFUL EDITION OF AE‐CAI

Welcome to this special issue of Wiley's IET Healthcare Technology Letters (HTL) dedicated to the 2024 edition of the augmented environments for computer‐assisted interventions (AE‐CAI), computer assisted and robotic endoscopy (CARE), and context‐aware operating theatres (OR 2.0) joint workshop. We are pleased to present the proceedings of this exciting scientific gathering held in conjunction with the medical image computing and computer‐assisted interventions (MICCAI) conference on October 6th, 2024 in Marrakech, Morocco.

Computer‐assisted interventions (CAI) is a field of research and practice, where medical interventions are supported by computer‐based tools and methodologies. CAI systems enable more precise, safer, and less invasive interventional treatments by providing enhanced planning, real‐time visualization, instrument guidance and navigation, as well as situation awareness and cognition. These research domains have been motivated by the development of medical imaging and its evolution from being primarily a diagnostic modality towards its use as a therapeutic and interventional aid, driven by the need to streamline the diagnostic and therapeutic processes via minimally invasive visualization and therapy. To promote this field of research, our workshop seeks to showcase papers that disseminate novel theoretical algorithms, technical implementations, and development and validation of integrated hardware and software systems in the context of their dedicated clinical applications. The workshop attracts researchers in computer science, biomedical engineering, computer vision, robotics, and medical imaging.

The 2024 edition of AE‐CAI | CARE | OR 2.0 was a joint event between the series of MICCAI‐affiliated AE‐CAI workshops founded in 2006 and now on its 18th edition, the CARE workshop series, now on its 11th edition, and the OR 2.0 workshop now on its 6th edition. This year's edition of the workshop featured 24 accepted submissions and reached more than 70 registrants, not including the members of the organizing and program committees, making AE‐CAI | CARE | OR 2.0 one of the best received and best attended workshops with more than a decade‐long standing tradition at MICCAI.

## 2 AE‐CAI—THE LONGEST‐LASTING SATELLITE EVENT AT MICCAI!

On the above note of “more than a decade‐long standing tradition at MICCAI”, it turns out that AE‐CAI, albeit several variations in name, has been running for a while now in some shape or form and is, in fact, MICCAI's longest‐standing workshop! Let us start with a historical note for those less familiar with our journey!

It all started in 2006 in Copenhagen under the name of AMI‐ARCS, which pointed to something along the lines of augmented medical imaging and augmented reality for computer‐assisted surgery, and it ran under that name for three more years, in Brisbane (2007), New York (2008), and London (2009). The 2010 edition (Beijing) was co‐hosted with the MIAR (medical imaging and augmented reality) conference. The workshop was then rebranded under its current name – AE‐CAI (augmented environments for computer‐assisted interventions) starting with its 2011 (Toronto) edition and it has been running under this name every year since, except for 2016, but let's leave that for another time.

## 3 AND THEN A BOOK WAS BORN …

Here is another memorable historical note that likely not too many know about: the *Mixed and Augmented Reality in Medicine* book, edited by Peters, Linte, Yaniv, and Williams and published by Taylor & Francis in 2019 was born out of the 2015 AE‐CAI workshop in Munich, Germany, when several of us decided to collect names and topics to perhaps one day put together a book – and then about three years later, that book was published! Thank you, Jackie, for shepherding the project and keeping us all in‐line!

## 4 REFLECTING ON THE LONGEVITY OF AE‐CAI

The longevity of the workshop series has been attributed to several stars aligning. One of these stars is the workshop organizing team – some of us have been involved since the beginnings, more than a decade ago, and somehow have not yet got tired of it (yet) – but the team is important, as it speaks for the peer review, organization and timeliness, and authors appreciate well‐organized and smoothly run conferences, and our team has been able to deliver on this, with exceptional, timely organization.

Another star aligning was timing: timing is often everything! In a way, AE‐CAI got lucky by starting early. Remember that back in 2006 when the first edition of AMI‐ARCS was hosted, MICCAI was less than 10 years old and workshops and satellite events were very scarce. Besides that, going back to its beginnings, in a way we also were lucky to get onto the AR/VR/MR wagon when we did back in the mid‐2000s, when augmented reality in medicine was buzzing. While it buzzed for a while, we were perceiving enough to realize that we needed to expand our scope and welcome more traditional CAI and image‐guided intervention work, much of which was no longer finding a home within the main MICCAI conference or other satellite events. So while we did not choose this specific area for a reason, I guess the group that first started the workshop were working in this area and wanted to bring the (small at the time) community together, which grew over time and our workshop grew alongside the community.

Another longevity star is the CAI community of MICCAI continuously seeking a venue for disseminating their research. Back in the mid/late‐2000s, augmented reality in medicine was gaining more popularity, and many CAI researchers started to venture into that domain. Nevertheless, despite its name, the workshop always welcomed and served as a home for a significant body of CAI research. While the CAI research used to be an integral part of MICCAI during its first 10 to 15 or even 18 years since its foundation in 1998, starting in the mid 2010s the focus of MICCAI, at least according to the perception of the CAI community and many CAI authors, started shifting further and further away from CAI, with only a select number of CAI papers being included in the program. As a result, many CAI researchers turned to the AE‐CAI workshop, that had been ongoing at the time, as their “preferred” CAI home within MICCAI. Hence, in a way, the AE‐CAI workshop series sort of “owes it” to MICCAI's perceived shift away from CAI, which helped the workshop grow stronger in response to the CAI community submitting really good quality work.

Another attractive feature to many authors has been the publication venue: starting with the 2011 edition, the AE‐CAI proceedings have been published in Springer's LNCS book series for five consecutive years, the same venue as MICCAI. Moreover, beginning with its 2017 edition, we have been fortunate to be invited by several peer reviewed journals to publish our proceedings as a special issue. This year's (2024) edition and its upcoming special issue in Wiley's Healthcare Technology Letters journal will constitute the 8th consecutive year of the AE‐CAI proceedings being published in a traditional peer‐reviewed journal venue.

The high standards of peer review that were instituted and maintained, especially with the proceedings being published in peer‐reviewed journal special issues, has been another attractive feature that has contributed to the longevity. The first stage of the review process is double‐blinded (same as MICCAI), with three external reviewers and 2 meta‐reviewers assigned to each paper. Following the first round of review, all authors must submit a detailed response to reviewers document and a revised manuscript, much like we do for journal articles. These revisions are then re‐reviewed, and if additional revisions are necessary, the paper is sent back to the authors for another round. We learned that to maintain quality, the peer review process cannot be rushed and the reviewer load needs to be maintained low. To that extent, we've been fortunate to have a reliable pool of reviewers, to whom we are very grateful. Each reviewer is only assigned 1–2 papers, so they can submit thorough reviews. This has helped us maintain the high quality of the published proceedings and a competitive overall acceptance rate.

Lastly, the generous sponsorship we have received over the years from Northern Digital Inc. (NDI) and Intuitive Inc. has allowed us to recognize several papers each year with paper awards has also been an attractive feature of the workshop series. We are greatly appreciative for this support.

In summary, the longevity of the AE‐CAI workshop series is really a result of the team's long‐term commitment and dedication to the community, the trust gained from the CAI community in response to maintaining high standards of peer review, the publication venue, and the paper award recognition thanks to our generous sponsors. Our consistent attendance and number of submissions / presentations year after year has made it clear that we have a fairly large community. Showing relevance, building and growing a community, and serving as a home for that community are critical factors for longevity and success. Then add to that some flexibility and creativity, and be ready to deliver: be organized, communicate consistently and timely, make the authors feel appreciated and respected and be grateful to the reviewers.

## 5 SHIFTING OUR ATTENTION TO THE PRESENT: THE 18^th^ EDITION OF AE‐CAI IN MARRAKECH, MOROCCO

The 2024 edition of the workshop was hosted as a single track, in person, event, where all accepted papers were featured as brief podium presentation as part of three sessions: *Augmented/Virtual/Mixed Reality Applications, Interventional Workflow and Skill Assessment, and Feature and Instrument Tracking for Image‐guided Interventions*. To further foster networking and discussion, following their podium presentations authors presented their work in a poster session.

Due to the limited time allotted to all satellite events at MICCAI 2024, we were unfortunately unable to include any keynote lectures in our workshop program, but we hope to resume this long‐standing tradition with the 2025 edition.

All manuscripts submitted to the joint AE‐CAI | CARE | OR2.0 2024 joint workshop were held up to journal standards, as the ultimate objective was to publish accepted work in this Special Issue of Wiley's Healthcare Technology Letters journal. This year's joint workshop received 32 manuscripts spanning a strong geographic representation from Europe, North America and Asia. The review process was rigorous and involved a double‐blind evaluation of each manuscript by two to four external reviewers. Following the first round of review, 6 manuscripts were accepted pending minor revisions, twelve manuscripts were accepted pending major revisions, four manuscripts were recommended for major revision and resubmission followed by another round of review, and the remaining 8 manuscripts were rejected. All authors were required to submit a response to reviewers for all manuscripts, along with a revised manuscript indicating all edits and changes in response to the reviewers’ critiques.

Once revised, all resubmitted manuscripts entered a second round of review conducted by the Program Committee, Workshop Chairs, as well as the Wiley's HTL Managing Editor and Editor‐in‐Chief to ensure that all reviewers’ critiques were properly addressed in the revised manuscripts and that the quality of the revised manuscripts was appropriate for journal publication. All author responses and revised manuscripts were revisited and assessed by the Joint Workshop Program Committee and Associate Editors, while also seeking the original reviewers’ opinion on the authors’ responses and revisions for manuscripts that underwent major revision and resubmission, as needed. Following the review process, twenty‐four manuscripts accepted for publication in this special issue and forwarded to the journal for production.

We would like to acknowledge the following manuscripts appearing in this Special Issue that have been recognized with an Outstanding Paper Award at the 2024 edition on the AE‐CAI Workshop in Marrakech!

Zijian Wu et al. Augmenting Efficient Real‐time Surgical Instrument Segmentation in Video with Point Tracking and Segment Anything.

Valentini Scarponi et al. FBG‐Driven simulation for virtual augmentation of fluoroscopic images during endovascular interventions.

Zahra Asadi et al. iSurgARy: A mobile augmented reality solution for ventriculostomy in resource‐limited settings.

## 6 THE 2024 AE‐CAI WOULD NOT HAVE HAPPENED WITHOUT

On behalf of the 2024 AE‐CAI | CARE | OR 2.0 Joint Workshop Organizing Committee, we would like to extend our sincere gratitude to all authors, presenters, and attendees for their scientific contribution, enthusiasm, and support. We also extend special thanks to all reviewers for providing detailed and timely critiques of the submitted manuscripts. We would also like to express our sincere thanks to our “boots on the ground” in Marrakech—Marta, Duygu, Elvis and Jonathan – for running the show on all of our behalf. We also acknowledge the support we received from the MICCAI 2024 Conference Organizing Committee and the MICCAI Workshop and Satellite Events Committee, as well as Wiley's HTL Editorial Office for supporting us in maintaining the high quality of the workshop through outstanding research contributions that fostered exciting discussions at the event.

Last but not least, we acknowledge our generous sponsors. We thank *Northern Digital Inc. (NDI)* for their continued support. Our sponsors’ generous contributions have enabled us to recognize our authors for their much‐deserved dedication and scientific enthusiasm through several best paper awards, as well as offset registration costs for student authors from developing countries. In closing, a big thank you on behalf of all awardees and registrants, and, once again, sincere congratulations to all award winners listed below!

## 7 IN CLOSING …

It's hard to believe it's been 18 years of AE‐CAI (and name variations), but then some of us on the team, although we have been attending it since 2006 or 2007, only go back as far as 2011 in terms or organization. Nevertheless, that's almost a decade and a half and we're still going strong. On this note, we would like to thank those who started this way back in 2006, who planted the AE‐CAI seed, and all the organizers who have been watering it year after year for 18 years. We would also like to thank all our authors and contributors going back nearly two decades, our many and dedicated reviewers for their timely effort year after year, our generous sponsors, and the MICCAI secretariat for their help and support!

We hope that you will enjoy reading this special issue and we look forward to your continuing support and participation in future editions of the AE‐CAI, CARE and OR 2.0 workshops. Their continued success demands our ongoing commitment and support, and we hope to welcome you all to the next edition of the workshop at MICCAI 2025 in Daejeon, South Korea. In the meantime, maybe we should consider planning big AE‐CAI party for its 20th edition – stay tuned!

